# High Throughput Method for Analysis of Repeat Number for 28 Phase Variable Loci of *Campylobacter jejuni* Strain NCTC11168

**DOI:** 10.1371/journal.pone.0159634

**Published:** 2016-07-28

**Authors:** Lea Lango-Scholey, Jack Aidley, Alexandra Woodacre, Michael A. Jones, Christopher D. Bayliss

**Affiliations:** 1 School for Veterinary Medicine, University of Nottingham, Nottingham, United Kingdom; 2 Department of Genetics, University of Leicester, Leicester, United Kingdom; Robert Koch-Institute, GERMANY

## Abstract

Mutations in simple sequence repeat tracts are a major mechanism of phase variation in several bacterial species including *Campylobacter jejuni*. Changes in repeat number of tracts located within the reading frame can produce a high frequency of reversible switches in gene expression between ON and OFF states. The genome of *C*. *jejuni* strain NCTC11168 contains 29 loci with polyG/polyC tracts of seven or more repeats. This protocol outlines a method—the 28-locus-CJ11168 PV-analysis assay—for rapidly determining ON/OFF states of 28 of these phase-variable loci in a large number of individual colonies from *C*. *jejuni* strain NCTC11168. The method combines a series of multiplex PCR assays with a fragment analysis assay and automated extraction of fragment length, repeat number and expression state. This high throughput, multiplex assay has utility for detecting shifts in phase variation states within and between populations over time and for exploring the effects of phase variation on adaptation to differing selective pressures. Application of this method to analysis of the 28 polyG/polyC tracts in 90 *C*. *jejuni* colonies detected a 2.5-fold increase in slippage products as tracts lengthened from G8 to G11 but no difference between tracts of similar length indicating that flanking sequence does not influence slippage rates. Comparison of this observed slippage to previously measured mutation rates for G8 and G11 tracts in *C*. *jejuni* indicates that PCR amplification of a DNA sample will over-estimate phase variation frequencies by 20-35-fold. An important output of the 28-locus-CJ11168 PV-analysis assay is combinatorial expression states that cannot be determined by other methods. This method can be adapted to analysis of phase variation in other *C*. *jejuni* strains and in a diverse range of bacterial species.

## Introduction

Simple sequence repeat (SSR) tracts are highly mutable sequences due to the potential for slip strand mis-pairing during DNA replication [1]. Slippage results in insertion or deletion of one or more repeats within the repetitive DNA tract. This combination of hypermutability and reversible mutations has driven evolution of SSR as a major mechanism of phase variation (PV) in several bacterial species [2,3]. PV describes a phenomenon of frequent, reversible alterations in expression of specific phenotypes [4]. These switches in expression are mediated by SSR located within the reading frame, core promoter or other regulatory sequences of a gene. The presence of multiple SSR-controlled phase-variable genes generates high levels of phenotypic variants within bacterial populations. Investigation of the fluctuations in population structure and adaptive benefits of these phenotypic variants requires detection of alterations in repeat number and correlation with expression state in multiple isolates from populations both prior to and post selection.

*Campylobacter jejuni* is a leading cause of human bacterial gastroenteritis, with contaminated meat products considered a main source of infection [5,6]. *C*. *jejuni* readily colonises the intestinal mucosa of a wide variety of wild and domestic birds and other animals. Infections in poultry are usually asymptomatic whilst human infection can result in significant inflammation and a profuse, bloody diarrhoea. As a commensal of poultry and a pathogen of humans, *C*. *jejuni* needs to rapidly adapt to fluctuations in host environments such as changing nutrient compositions and appearance of innate/adaptive immune effectors. Further selective pressures are caused by transmission through genetically and immunologically variable host populations and exposure to bacteriophages. *C*. *jejuni* is likely to use PV as a major mechanism for adaptation to these selective pressures.

Mononucleotide repeat tracts consisting of seven or more cytosine or guanine bases are an unexpected feature of the AT-rich genomes of *C*. *jejuni* [7]. These SSR are the main mechanism of PV in this species and an analysis of four genome sequences indicated the presence of 12 to 29 tracts per genome [8]. The majority of these loci are predicted to encode enzymes involved in modification of surface structures (i.e. lipooligosaccharide, flagella and capsule) but a few encode surface proteins or restriction enzymes [9–12]. There are 29 polyG/polyC tracts in *C*. *jejuni* NCTC11168. The majority (n = 23) of these tracts are located within the main part of the reading frames whilst the others are at the 3' end of the reading frame (n = 1; *cj0045*), in pseudogenes (n = 2; *cj0046* and *cj0676*), or in intergenic regions (n = 3; *cj0565*, *cj0742* and *cj1321*) [9]. The tracts in the reading frame mediate ON or OFF switches in gene expression as shown for a sub-set of genes using reporter constructs or antibodies against the encoded protein or modified epitope [8, 13].

An important aim has been to understand the contributions of PV to specific phenotypes and to more generic behaviours such as colonisation of host animals. Some limited experimental characterisation has been performed on individual phase-variable genes, including locking the reading frame into an ON or OFF state [14–16]. A more generic approach has been to analyse the whole population by PCR using fluorescent primers and fragment analysis on an auto sequencer. The major and minor tract lengths are estimated from the relative sizes of peaks [17–19]. This approach is limited by the slippage that is known to occur in repetitive tracts during PCR [20]. An alternative approach is to perform next generation sequencing (NGS) and estimate numbers of variants from sequences spanning a repeat tract. However, NGS is not as high-throughput as PCR and the error rate of NGS increases as a function of repeat number, hence NGS has not yet been widely used to analyse PV [21]. Furthermore, while both of these approaches have utility for determining the PV states for individual genes neither can be used for determination of combinatorial expression states.

The presence of multiple phase-variable genes in *C*. *jejuni* genomes raises the potential for combinatorial effects particularly where genes are involved in modification of the same macromolecule [9]. In order to investigate this phenomenon, the tract lengths of each phase-variable gene within individual cells need to be determined. Single colonies, derived from a population by plating out serial dilutions, can be utilised as surrogates of single cells under the assumption that switches in the repeat tract are infrequent during growth of the colony. As switching rates are ~1x10^-3^ mutations per division [8], the major tract length of each gene in each colony will be the same as the initial, derivative single cell. The major tract length can then be determined for colony lysates by PCR and fragment analysis. A multiplex PCR and fragment analysis assay was previously developed for six of the polyG/polyC tracts of *C*. *jejuni* strain NCTC11168 and used to determine expression states for six genes in samples from *in vitro* and *in vivo* passage experiments [8]. A manual analysis protocol was utilized for assessing tract length and expression state from the fragment sizes output of the fragment analysis assay. Expression states were determined for 30 colonies derived from each population as well as DNA extracted from the total population. Estimates of the major tract length and expression state were similar for each sample indicating that the method was robust for determining expression states and was utilized for analysis of combinatorial expression states [8].

A scale up of the six-gene PV assay to incorporate all 28 phase-variable loci of *C*. *jejuni* required navigation of a series of complicated issues. Some of the phase-variable loci contain identical sequences in and around the repeat tracts necessitating development of locus-specific primers and careful design of multiplex PCR reactions. A 28 multiplex fragment analysis assay was required in order to minimise cost and maximise throughput. Similarly, high throughput and accuracy necessitated an automated extraction process for conversion of fragment analysis data into tract lengths and expression states. This paper describes a rapid and accurate 28-locus-CJ11168 PV-analysis assay whose data outputs include user-friendly formats readily accessible to further analysis in downstream applications.

## Materials and Methods

### Part I—Multiplex PCR amplification of 28 phase-variable loci

#### Bacterial DNA extraction

*C*. *jejuni* colonies were grown on Campylobacter blood-free selective agar plates (CCDA, Oxoid) for 48 to 72 hours at 37°C in 5% oxygen, 10% carbon dioxide and 85% nitrogen. For DNA extraction, single colonies were re-suspended in 100 μl molecular-grade water in 0.5 ml PCR tubes or 96-well plates and heated to 95°C for 5 min. Tubes were then briefly vortexed before centrifugation for 2 min. The aqueous part (containing bacterial DNA) was transferred into a 96-well plate and stored at -20°C.

#### Primer design

Primers were designed to generate PCR products spanning each repeat tract (Table 1) and having sizes between 80 and 470 base pairs in length (Table 2). One primer in each primer pair was labelled with a fluorescent dye (FAM, VIC or NED; Table 1). Non-labelled and FAM-labelled primers were supplied by Eurofins MWG or SIGMA, VIC- and NED-labelled primers were supplied by Applied Biosystems.

**Table 1:** Phase-variable genes and repeat tract specific PCR primers.

Gene	Gene size (bp)	Location of Repeat Tract	In-frame Repeat Number^1^	Primer name	Sequence (5' → 3')
*cj0031*	3,732	+2,572	9G	cj0031-fwd-FAM cj0032-rev	GGCTTTGATCTCATCATCGG GCAAAGCTTCCCCATATCCT
*cj0045c*	723	+709	11G^2^	cj0045-fwd-FAM cj0045-rev	TTTTACACTAGAACACAGAAG CCTTAAAGTGCGAAAAATGTG
*cj0046*	1,407	+610	(11G)	cj0046-fwd-NED cj0046-rev	TCAAATACTGCAAGAGCAGG TAGAAGCATTAGGCGTGG
*cj0171*	744	+243	8G	cj0171-fwd-NED cj0171-rev	TGGTTGTGGAAATGGAGTGC GCTCCTTCATTGCATAGTTC
*cj0275*	1,251	+694	8G	cj0275-fwd-NED cj0275-rev	ATTACTCGTGATGTAAGTGG AAACCTACAACTTTATCTCC
*cj0565*	729	-58	(10G)	cj0565-fwd cj0565-rev-VIC	AATTTCACTTCCCCCTTGACT TTTTGCAACATCGCGTAGAA
*cj0617*	609	+559	10G	cj0617-fwd-VIC cj0617-rev	TGGTATAATGCAAGCTATGG AAATCAATACTCCAAGGAGC
*cj0628 (capA)*	3,432	+501	11G/10G^3^	CapA-fwd-FAM CapA-rev	TATTTCTAATGATGGGCAAC GAACGAACATTTACACCCAT
*cj0676*	1,656	+854	10G	cj0676-fwd-NED cj0676-rev	ATGCTTATTCCTAGTGCCTG TGCATTTAAACCCAAAGAATCC
*cj0685c*	1,350	+877	9C	cj0685-fwd-FAM cj0685-rev	GATAGCGAATATAACCTCTAAATTC GAAGAAATCCGCCAATCAAAG
*cj1139c*	909	+330	8G	cj1139-fwd-VIC cj1139-rev	GCAACTTCACCTTATATC TAAATTCTTTGTTGTTGTATTTTCC
*cj1144c*	843	+294	10G	cj1144-fwd-NED cj1144-rev	GATGTTGTGATTCTTG GTAGCAGCGTTTAGTG
*cj1295*	1,305	+143	9G	cj1295-fwd-NED cj1295-rev	TTCCTATCCCTAGGAGTATC ATAGGCTTCTTTAACATTCC
*cj1296*	792	+309	10G	cj1296-fwd-NED cj1296-rev	ATAAAGTGCATTCTAAAGGC CAGCAAAGGAAAAAATAGGG
*cj1305c*	1,215	+579	9G	cj1305-fwd-VIC cj1305-rev	CAACTTTTATCCCACCTAATGGAG AAAGCCGAACCCGAATTATC
*cj1306c*	1,224	+579	9G	cj1306-fwd-NED cj1306-rev	TTTATTCCTTCGCGTGGAGA AAAAATGATCGCCCTGCAT
*cj1310c*	1,212	+579	9G	cj1310-fwd-FAM cj1310-rev	GAACAAATTATTCCTCTTATAG TCGAAATAAAATTCCCCTTGA
*cj1318*	1,947	+167	11G	cj1318-fwd-FAM cj1318-35-rev	TCCGTGCGTCCTTCTTTTGGAC GTTTGCAACTCTTTAATGGG
*cj1321*	540	-37	(10G)	cj1321-fwd-VIC cj1321-rev	AAAAAGGAATGATGCGTTGC CCCGCTCCTATGATGATGAC
*cj1326*	672	+252	9G	cj1326-fwd-FAM cj1326-rev	CTTTTGGAATAGATATAGTTCC TTAGAGGTATGTAGTAAAGAC
*cj1335*	1,944	+168	11G	cj1335-fwd-NED cj1318-35-rev	CACAATTGGTTTATCCAAGG GTTTGCAACTCTTTAATGGG
*cj1342c*	1,239	+560	9G	cj1342-fwd-FAM cj1342-rev	TTGGCAATCGTCCTCAAACC GCCAAATGCGCTAAATATCC
*cj1420c*	771	+393	9G	cj1420-fwd-FAM cj1420-rev	GCTAGTTCTTTCCATTGGAC CTACAATGTGGCGAGGATTC
*cj1421c*	1,836	+87	9G	cj1421-22-fwd cj1421-rev-VIC	TTGGGTATTTAAGTTGGGGAAA TCAAAACCCATCTTTATCATTTTCT
*cj1422c*	1,875	+87	9G	cj1421-22-fwd cj1422-rev-NED	TTGGGTATTTAAGTTGGGGAAA AATGATTTTGCTTTGCAGGAA
*cj1426c*	849	+294	10G	cj1426-fwd-FAM cj1426-rev	TATAGCCGATCCACAAGG GATATAACTTTGCCCGCCAC
*cj1429c*	924	+291	10G	cj1429-fwd-FAM cj1429-rev	ATGGAGATGGTGGTTATGTG ACTATCCGAAACACCCAAAG
*cj1437c*	1,101	+876	9G	cj1437-fwd-VIC cj1437-rev	GTGCTAGGATGGAATTTGTG CAAACAAGGTGAAAACCTCC

^1^Values in brackets indicate the arbitrary number of repeats coded as ON because the repeat tract is intergenic or within a pseudogene.

^2^G11 would allow the translation to extend by another 15 amino acids, overlapping the start of *cj0044c*; G9 and G10 (consensus) have little effect.

^3^*capA* fragment varies between 11168 strains due to variation in the polyT tract immediately upstream of polyG tract (6T in the lab strain and the hypermotile 11168H strain, 5T in the chicken-adapted 11168ca strain).

**Table 2:** Multiplex PCR primer mixes.

Primer mix	Locus	Dye colour^1^	PCR product size^2^
**A**	*capA* *cj0031* *cj0045* *cj0685* *cj1326* *cj1342*	Blue Blue Blue Blue Blue Blue	457 221 280 128 165 392
**B**	*cj1318* *cj1420* *cj1426* *cj1429* *cj1437*	Blue Blue Blue Blue Green	332 109 209 89 275
**C1**	*cj0275* *cj1296* *cj1306*	Yellow Yellow Yellow	215 104 304
**C2**	*cj0171* *cj1144* *cj1335*	Yellow Yellow Yellow	282 173 337
**D**	*cj0565* *cj0617* *cj1139* *cj1305* *cj1321* *cj1421*	Green Green Green Green Green Green	357 167 200 220 149 287
**E**	*cj0046* *cj0676* *cj1295* *cj1310* *cj1422*	Yellow Yellow Yellow Blue Yellow	353 206 159 365 399

^1^Dye colour as annotated in output files from PeakScanner^™^ although yellow is displayed as black in this program.

^2^PCR product size, as determined from the *C*. *jejuni* NCTC11168 genome sequence (7).

Primers were designed so that the products of each primer pair could be distinguished by either fluorescent label or size of PCR product (Table 2). Any PCR products that gave overlapping peaks during fragment length analysis were clearly distinguishable by different dye colours. Three pairs of genes (*cj1295* and *cj1296*; *cj1305* and *cj1306*; *cj1421* and *cj1422*) and a group of three genes (*cj1310*, *cj1318* and *cj1335*) have regions of identical or high sequence identity. Gene-specific primers were designed for *cj1295*, *cj1296*, *cj1305*, *cj1306* and *cj1310* genes. These primers differed at 1 or more positions including the 3' terminal nucleotide. For *cj1421-cj1422* and *cj1318-cj1335*, a common unlabelled primer was combined with a fluorescently-labelled gene-specific primer whose fluorescent tag differed for each gene of the two paired loci, so that similarly-sized PCR products could be easily distinguished.

Repeat numbers for control samples and for validation were determined by dideoxy sequencing of PCR products. These products were generated by amplification with non-labelled versions of the fragment analysis primers or, for the loci with short PCR products, additional primers located further from the repeat tract. DNA sequencing reactions were performed with Phusion high-fidelity DNA polymerase (Thermo Fisher).

#### Design of multiplex PCR reactions

A series of six PCR reactions were designed to amplify between three and six loci (Table 2). Primers for genes with high sequence identity or using a common primer were assigned to different reactions in order to prevent false-priming or competition between amplifications. Reaction mix C was designed as a six locus reaction but was split into two reactions (C1 and C2) due to inefficient amplification of some genes. Primers were stored as combined mixes of six to twelve primers at a concentration of 2 μM for each oligonucleotide.

#### PCR amplification and GeneScan assays

PCR reactions were performed using GoTaq polymerase (Promega) or KAPA Taq (KAPA Biosystems) with standard conditions (Table 3) in 96-well PCR plates sealed with an adhesive film (Thermo Fisher, AB-0558). A sub-set of samples from each plate were checked on 2% TAE agarose gels to confirm amplification. PCR products from each reaction were pooled together and an A-tailing reaction was performed (Table 3) to ensure uniform addition of an untemplated adenine to all PCR products. Samples were stored at -20°C, as required, between steps.

**Table 3:** PCR amplification and A-tail reactions.

Step	Reaction mix for GoTaq (Promega)	Reaction mix for KAPA *Taq* (KAPABiosystems)	Reaction in PCR cycler
**Multiplex PCR**	5x Buffer—2 μl dNTPs (10mM)—0.5 μl MgCl_2_ (25mM)—1.2 μl Primer mix (2 μM)—1 μl GoTaq polymerase—0.1 μl DNA template—1 μl Water—to 10 μl	10x Buffer A—1 μl dNTPs (10mM)—0.5 μl MgCl_2_ (25mM)—1.8 μl Primer mix (2 μM)—1 μl KAPA Taq polymerase—0.1 μl DNA template—1 μl Water—to 10 μl	94°C—5 min 25 cycles: 94°C—30 s 50°C—30 s 72°C—60 s 72°C—5 min 10°C—hold
**Pooling of PCR products and A-tail reaction**	**A-tail mix:** 5x Buffer—0.8 μl MgCl_2_ (25mM)—1 μl GoTaq polymerase—0.05 μl Water—to 4 μl	**A-tail mix:** 10x Buffer A—0.4 μl MgCl_2_ (25mM)—0.4 μl KAPA Taq polymerase—0.05 μl Water—to 4 μl	Transfer to a new 96-well plate: - 4 μl A-tail mix - 2 μl each multiplex PCR reaction (total volume 16 μl) 72°C—45 min

Protocol for amplification of 28 PV sites by multiplex PCR in 96-well plates, including the A-tailing step before mixing of samples with Size Standard for GeneScan analysis.

### Part II—Multiplex GeneScan and fragment length analysis

**Fig 1:**
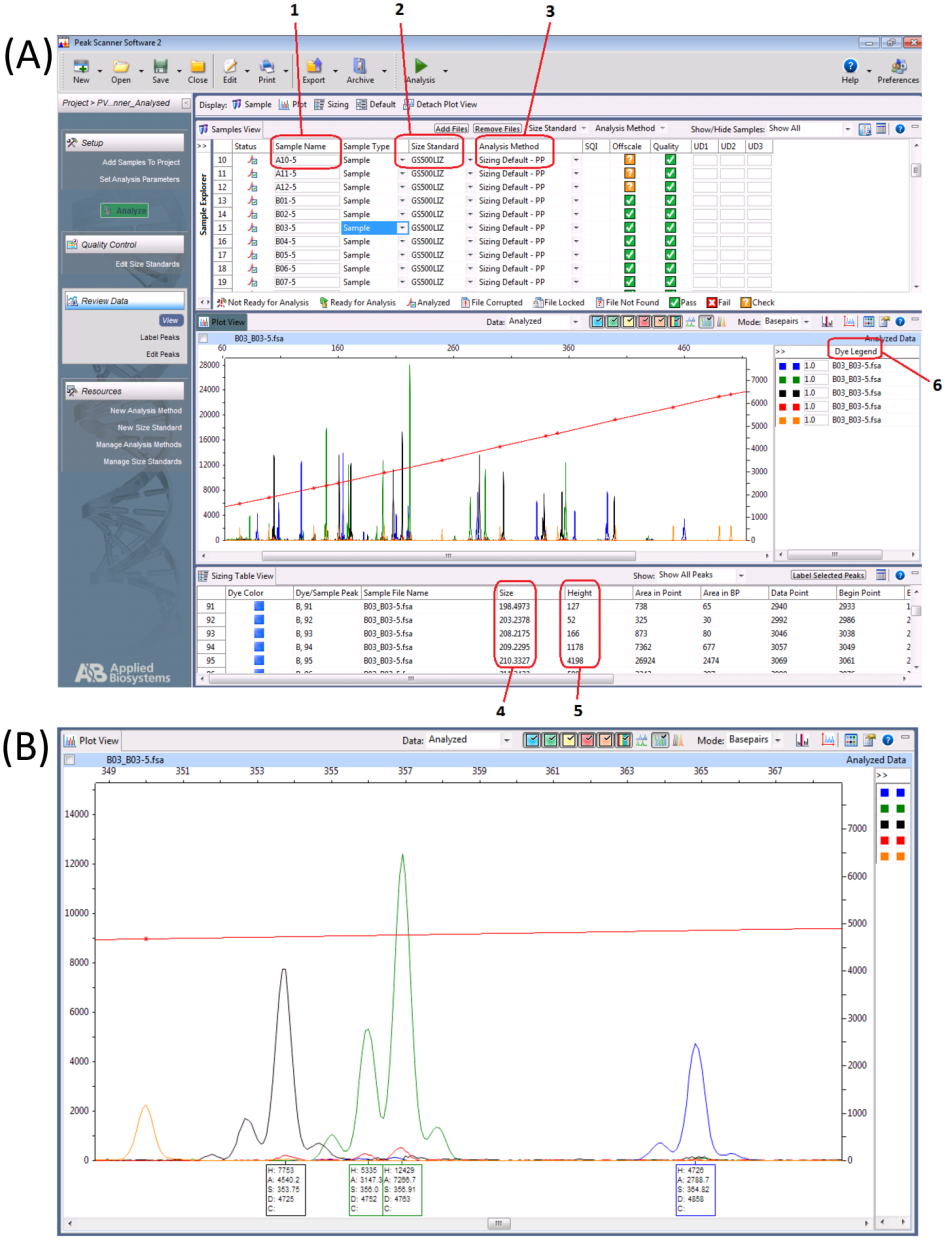
Analysis of multiplex fragment analysis data in PeakScanner. (A) PeakScanner application layout. Labels 1–6 refer to: 1, Sample Name, well location in the 96-well plate; 2, Size Standard, name of size standard as selected from the drop-down menu; 3, Analysis Method, analysis method name, e.g. PP—Primers Present, assumes primers have not been removed from the sample; 4, Size, length of PCR fragment for a specific peak in base pairs; 5, Height, height of each peak indicating fluorescent dye signal strength; 6, Dye Legend, displayed dye colours and relative scale. (B) Enlarged section of Panel A showing how similarly-sized PCR products can be distinguished using different dye colours and the occurrence of flanking peaks around a major peak.

A 1.5 μl aliquot of pooled A-tailed PCR products for a specific colony was added to 9.25 μl of deionized formamide and 0.25 μl of a GeneScan ladder (i.e. GS500LIZ or GS600LIZ). Test samples were arrayed in 96-well PCR plates with PCR and fragment analysis control samples (see below). Samples were subject to capillary electrophoresis on an ABI3100 analyzer. Output files (*.fsa) generated by the autosequencer were loaded into Peak Scanner^™^ software. Determination of fragment size was performed using default settings, with GS500LIZ or GS600LIZ specified as size standard and ‘PP—Primers Present’ in the ‘Analysis Method’ column. An example of PeakScanner^™^ graphical view is shown in Fig 1A.

### Part III—Automated determination of repeat number and expression state

#### Data extraction from PeakScanner using PSAnalyse

A complete output data set, including detailed information about all the peaks from each sample in the 96 well plate, was extracted from PeakScanner^™^ as a tab-delimited text file using the ‘Export Combined Table’ command. We have developed a Perl script (PSAnalyse) that will accept this output file of PeakScanner^™^ and analyse each sample to determine the fragment length, peak height, repeat number and expression state of the major peak for each of the 28 phase-variable loci of *C*. *jejuni* strain NCTC11168. Additionally the script will output the ratio of the major peak to flanking peaks. The repeat numbers and ON/OFF states for each PV locus are generated by comparison to control samples with known repeat numbers and expression states. Control samples were located on the same plate as the test samples. For ease of use, we also developed a user-friendly C# frontend allowing use of the script via text boxes/drop-down menus as an alternative to entry from the command line.

After input of the tab-delimited text file into PSAnalyse, the user sets cut-offs for peak ‘height’ (normal setting for efficient PCR reactions is 1,000), ‘ratio to flanking peaks’ (default setting is 1.5), ‘tract error’ (distance of observed peak size from expected peak size; allows for small well-to-well variations in migration of PCR products; default setting is 0.33 bp) and ‘scan width’ (distance from expected peak size for identification of a peak; default setting is 3.5 bp). The user then identifies the location of the control samples and assigns the matching peak set file, which contains the expected fragment size and fluorescent tag for each locus, and enables the program to assign each peak to a specific locus. The ‘peakset’ files also defines the repeat number (or numbers) associated with an ON expression state. The user also sets the calibration file (calib file), which contains the previously determined repeat numbers for the specific control sample present in the control well. The user can also input a CSV file with the names of each sample for each well of the analysed plate otherwise sample names are based on the tab-delimited text file exported from PeakScanner^™^. When run, PSAnalyse generates a series of output files (Table 4) containing combined and separated data for each sample. Note that expression states are coded as 0 for OFF and 1 for ON while datapoints that fail quality control checks are indicated with a ‘?’ and missing data are shown with an ‘N’.

**Table 4:** Output files for PSAnalyse.

Output file name	Format	Information
Input_name.details.csv	CSV	Detailed output that extracts and displays relevant data from PeakScanner (Sample name, Size, Height, Area, Size of Prev, Area of Prev, Size of Post, Area of Post) and also calculates and displays ‘Ratio to Prev’, ‘Ratio to Post’, ‘Tract Length’ and ‘Score’– 0 or 1 –based on tract length.
Input_name.tracts.csv	CSV	Contains tract length data for the 28 PV sites for each sample in a 96-well plate as a single table. ‘N’ is used for missing datapoints and datapoints that are identified as uncertain have the suffix ‘?’.
Input_name.scores.csv	CSV	Contains ON/OFF scores (1 or 0 respectively) only for all 28 PV sites for each sample in a 96-well plate as a single table. ‘N’ is used for missing datapoints and datapoints that are identified as uncertain have the suffix ‘?’.
Input_name.out.html	Html	Graphical visualisation of Output results for all 28 PV sites in each well (sample); allows easy identification of problematic results as these are coloured in red.

#### Accession number

PSAnalyse and the control and test sequencing data are available on Dryad with accession number:- doi:10.5061/dryad.k9b6f.

## Results and Discussion

Analysis of the multiple phase-variable genes of *C*. *jejuni* strains requires detection of indels in hypermutable polyG tracts and of the combinatorial effects of expression states. We have therefore developed a high throughput method—the 28-locus-CJ-11168 PV analysis assay—that involves multiplex PCR and fragment analysis of 28 PV loci from single colonies of *C*. *jejuni* strain NCTC11168. The method involves semi-automated analysis of PCR fragment sizes, repeat numbers and ON/OFF states for each locus. The method can be split into three stages:- (1) amplification of 28 PV sites by multiplex PCR using fluorescently labelled primers; (2) fragment length analysis by capillary electrophoresis and PeakScanner^™^; and (3) automated calling of repeat numbers and assignment of ON/OFF states for each gene using a custom script, PSAnalyse.

### Development and testing of the multiplex PCR and GeneScan assay

The crucial features of the primer design for the multiplex PCR assay were:- high specificity of at least one primer within a pair for the target locus; and ensuring that no two PCR products had both the same or similar size (<15 base pairs difference) and the same fluorescent label. This design ensured that every PCR product was distinguishable by size, dye colour or both. The 28-locus-CJ-11168 PV analysis assay utilised this approach to encompass 28 of the polyG/polyC tracts present in this strain (Table 1). One of the intergenic repeats, located at the 3’ end of two convergent genes (*cj0742* and *cj0743c*), was excluded in order to reduce the potential for generating overlapping PCR products and due to the limited potential for this repeat to influence gene expression. The primers for *cj1305* were observed to have additional binding sites in *cj1306* and a downstream region resulting in >4 kbp product. Amplification was, however, inefficient and did not influence production of the expected 219 bp fragment for the SSR within *cj1305*.

To validate the assay, a series of lysates were prepared from single colonies of *C*. *jejuni* strain NCTC11168. These lysates were analysed using the multiplex PCR/fragment analysis assay and repeat numbers were assigned for each gene using PSAnalyse. A random set of these lysates were then re-amplified using locus-specific primers without fluorescent labels (2–13 per locus) and then these PCR products were subject to dideoxy sequencing (S1 Table). The sequence data was analysed by BLAST to confirm that the correct locus had been amplified and then repeat number was determined from observation of the trace files. For the genes with regions of high identity (*cj1295* and *cj1296*; *cj1305* and *cj1306*; *cj1421* and *cj1422*; and *cj1310*, *cj1318* and *cj1335*), there was only a limited number of sequence differences and these were examined in the trace files to confirm locus specificity. Due to the short length of some of these PCR products, tract length was validated by PCR amplification using primers located in non-identical flanking sequences and sequence analysis of the whole PCR fragment. In all cases, products were obtained from the expected locus and the observed repeat number matched the predictions from the 28 locus PV-analysis assay (S1 Table).

### Development of multiplex PCR and GeneScan assays

Primers for multiple loci were combined in order to reduce the number of required PCRs (Table 2). Initially five primer mixes were designed to amplify five or six loci each. Primers for genes with high sequence identity or using a common primer were assigned to different reactions in order to prevent false-priming or competition between amplifications. Small differences in PCR efficiency were accommodated by setting the minimum peak height for detection of fragments by PSAnalyse below the height for the least efficient PCR reaction. Four of the primer mixes generated the expected products at similar efficiencies. Reaction mix C exhibited low PCR efficiencies for some loci. PCR efficiency was improved by splitting mix C into two reactions (C1 and C2) of three genes.

PCR products from the six PCR reactions were combined into a single sample and arrayed on a 96-well PCR plate. Two types of controls were included on this plate. A set of PCR products amplified from a genomic DNA preparation with known repeat numbers (i.e. determined by dideoxy sequencing) and a previously prepared set of fluorescently-labelled products. The former controls for variation in the efficiencies of PCR reactions and the latter for plate-to-plate variations in the migration of products on the autosequencer. Samples were subject to electrophoresis on an autosequencer.

### Development and implementation of a custom script for automated PV state analysis

Each well of the fragment analysis plate can be analysed and manually interrogated in PeakScanner^™^ to extract PCR fragment size. Comparison of these values to the control data enables the repeat number and expression state to be determined. As this process is laborious and subject to error, an automated programme, PSAnalyse, was developed to extract data from PeakScanner^™^ output files. These data sets contain multiple, spurious peaks as a result of non-specific primer binding, primer-dimers, and background ‘noise’ during detection. In order to extract only the relevant information, the program was designed to search for fragments within specific regions of the scans performed by the autosequencer. Thus the basic principle of PSAnalyse is recognition of the highest peak of the correct colour (i.e. fluorescence) within a user-defined range (e.g. 3 nucleotides plus or minus) of the expected size (pre-set values contained in Peakset files). The features of this major peak and of the flanking peaks (size, height and area under the peak) are extracted and compared (see Fig 1B for an example of major and flanking peaks for a locus). Peaks that exceed user-defined cut-offs for height and ratio to flanking peaks are accepted as the observed peak for that locus otherwise peaks are flagged as problematic (allowing for the user to re-examine the PeakScanner^™^ trace file). The repeat number and gene expression state for each locus are assigned based on size relative to control samples.

The ‘scan width’ setting enables detection of phase-variants that have shifted by a number of nucleotides from the starting number for a given experiment. The polyG/polyC tracts of *C*. *jejuni* strain NCTC11168 were observed to increase or decrease by one nucleotide during examination of PV rates without selection and by up to 4 nucleotides during selection experiments [8]. As discussed below, the scanning range can be widened but must prevent overlaps between loci labelled with the same dye colour.

A common source of flagged errors for individual tracts was for the expected size of the PCR fragment to exceed the limit by up to 0.2 bp (note the PeakScanner reports fragment sizes to four decimal places). These errors arise due to differences in migration of fragments and could be corrected by inspecting surrounding samples to determine if a sample was aberrant or part of a trend for a series of samples. Another frequent observation was for differences between odd and even wells in the first plate of a series when using the GS500LIZ size standard (resulting in data flagged as an error due to observed peaks being 0.5 bp larger than control peaks). This difference was attributed to warming of the plate and to use of a 48 capillary machine so that odd wells of the first plate were injected whilst the plate was still cold. Inclusion of control samples in both odd and even wells controlled for this difference. This problem was eliminated in later analyses by switching to the GS600LIZ size standard, which is not subject to variations in fragment size. Another problematic observation was of the presence of occasional spurious peaks in wells containing no samples. The majority of these peaks could be eliminated by setting the minimum peak height to 1,000. This setting resulted in loss of data from weak PCRs and recovery of this data had to be assessed against the potential for inaccurate sizing of a small sub-set of peaks.

The expression states for each locus were based on an analysis of the whole genome sequence for *C*. *jejuni* strain NCTC11168 (Table 1) [7]. For the genes with tracts located in the reading frame, the ON expression state was determined as the repeat number associated with an intact reading frame. In general, a single repeat number was provided in the peakset file as a determinant of the ON state. In some cases, variants were observed that had switched by three nucleotides to the next available ON state but were being reported as OFF by our early version of PSAnalyse. Additional columns were added to the peakset files for the other expression states and PSAnalyse was modified to search for these expression states or to treat as ON all values differing from the given ON state by a multiple of three nucleotides/repeats.

An exception to our generalised approach to expression states was observed for the *capA* gene. Ashgar *et al*. [15] had reported (and was confirmed by dideoxy sequencing) that variants of NCTC11168 differed by one nucleotide within the A-tract, that is adjacent to the G-tract within the *capA* reading frame, resulting in an alteration in the reading frame. Peakset files for different variants were utilised with the ON repeat number for *capA* being either 10 or 11 nucleotides. Another exception is that the ON number of repeats was arbitrarily set for *cj0045* (the tract in this gene is located at the 3’ end of the gene), pseudogenes and intergenic repeats. Coding of some of the variation for these loci with the simplified binary code facilitated further analyses.

### Comparison of single colonies versus total DNA extracts for estimation of ON state proportions in a mixed population

Previous investigations reporting ON/OFF states of individual PV genes by the fragment analysis method have analysed the relative ‘area under peak’ for each amplified PV site. In this approach, the DNA template was the total DNA extracted from the whole population of a given sample. This method is, however, subject to error due to slippage during PCR amplification of long homopolymeric tracts [20].

We compared how data generated from analysing individual colonies differed from analysis of total DNA extracts of a population. A set of 16 samples were selected for this analysis (these samples were isolated using selective media from *C*. *jejuni*-infected birds at 52 days post-inoculation; Lango-Scholey *et al*., in preparation). DNA was extracted from large sweeps of confluent growth or >100 colonies (‘total’ DNA) and in addition from 30 individual colonies derived from dilutions of the same population. Tract lengths for individual colonies were assigned based on the major peak using PSAnalyse and then a % ON value was determined by dividing colonies with an ON repeat number by total number of colonies. For ‘total’ samples we calculated relative areas under peaks for tracts of different sizes and representing different ON and OFF states and then calculated the % ON value by dividing the area for ON peak(s) by the total area under all peaks (note that most samples contained one major peak and two minor peaks; see S1 File). The difference between the two analysis methods was assessed by subtracting the two values.

**Fig 2:**
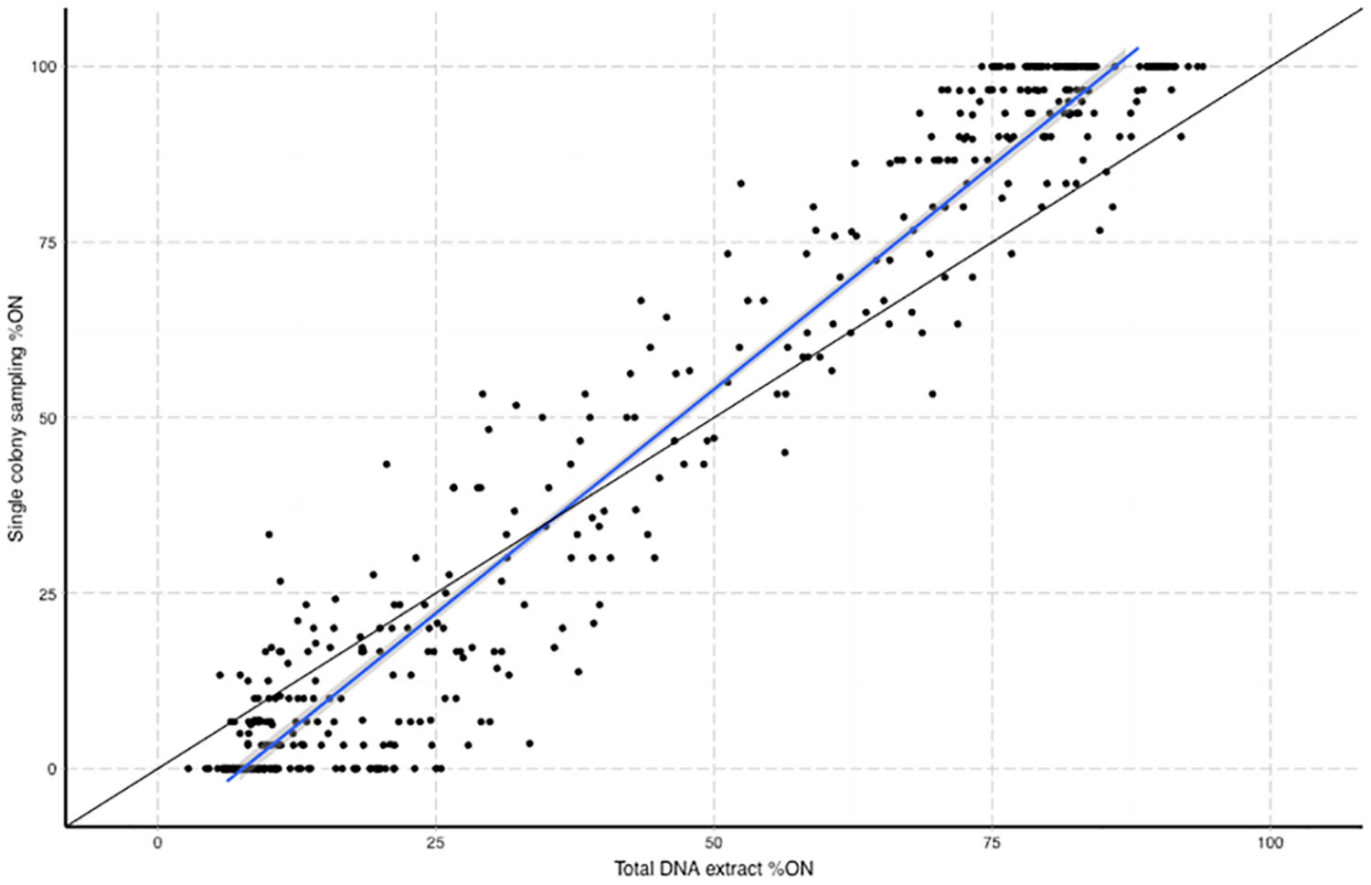
Comparison of two analysis methods for determining the percentage of cells in a population with a gene in an ON state. The percentage of ON variants in a population was determined by PCR-based fragment analysis of either the relative proportions of peaks obtained using a total DNA extract of the population (x-axis) or from analysis of up to 30 single colonies obtained from serial dilutions of a population (y-axis). The analysis was performed on 16 populations for 28 phase-variable loci. Each circle represents one of the 448 measurements. Black line, line for a 100% correlation between each method. Blue line, linear regression line with 95% confidence interval indicated by shaded area.

A set of 448 values for % ON (28 genes in 16 samples) was obtained by both the whole population (i.e. a sweep) and single colony analysis methods. Fig 2 shows the linear regression line (blue line) between % ON values for the two sampling methods. Divergence from the expected correlation (black line) exhibits two opposing trends, under- or over-estimation of %ON by the total colony method relative to the other method at high and low %ON states, respectively. The intersection between the lines for observed and expected values occurs at ~33%, which corresponds to the known relationship between ON and OFF states of 1:2 for intragenic SSR-mediated PV. Examination of values for individual genes (S1 Fig) did not show any consistent trends indicating that the differences are independent of gene function. As an example, the total population method detected small peaks flanking the main peak of *cj0275* and predicted that ~9% of cells in these populations were in the OFF state (data not shown). However, the only repeat number detected in 390 single colonies was G8 (ON) and indeed no variants have been detected so far during analysis of >2,000 colonies isolated from infected birds (Lango-Scholey *et al*. in preparation). Similarly, for the 11 birds in which *cj1429* was mainly in an ON state (i.e. G10), the total population analysis predicted that 77% of colonies were in an ON state whereas 93% (279 out of 299) of the single colonies were in this state. These results suggest that the detection of peaks correlating with OFF repeat numbers in the total population analysis were generated by PCR slippage rather than representing actual phase variants. Thus, PCR slippage appears to lead to inaccurate assessments of the proportion of cells in an ON or OFF state when PCR and fragment analysis of total DNA preparations are utilised for assessment of PV states in a population. The greatest differences are observed in populations exceeding 99% ON or 99% OFF (with values ranging up to 25% lower).

### Analysis of PCR slippage during investigation of repeat numbers in single colonies

**Fig 3:**
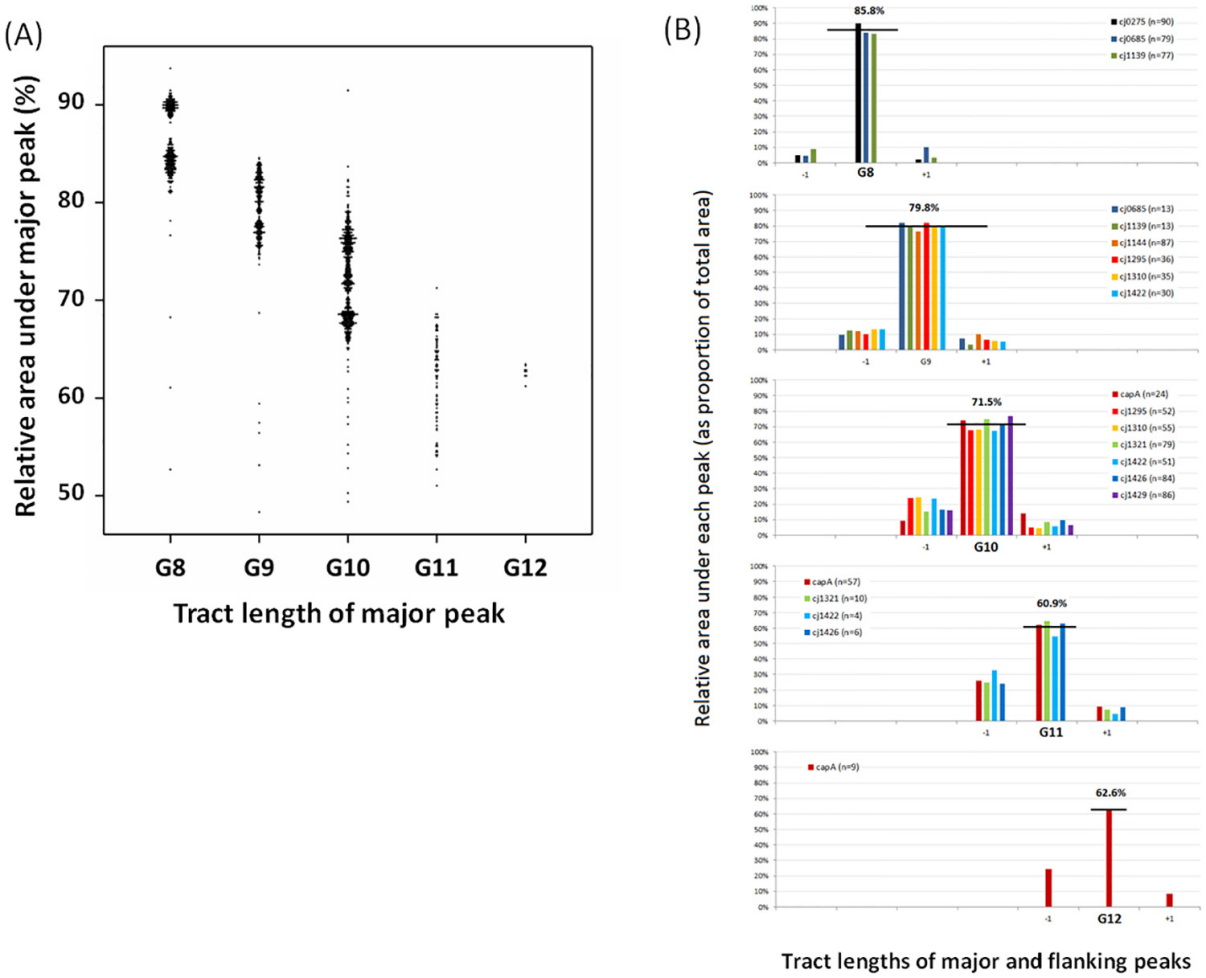
The effect of repeat number on PCR slippage as detected by fragment analysis of individual colonies with an ON number of repeats. Fragment length analysis was performed for 11 repeat tracts on a set of single colonies (n = 90). The relative area under each peak (i.e. the flanking peaks and the major peak) was calculated by dividing the area under the peak by the total area under all peaks for that specific locus and sample. These values were multiplied by 100 to obtain percentage values for the relative area under each peak. (A) Dotplot of individual values for the major peak of each locus separated by tract length. The number of loci analysed for each tract length were as follows:- G8, 257; G9, 214; G10, 431; G11, 77; G12, 9. (B) Average values for each locus. Peaks for each locus were separated by the numbers of repeats in the polyG tract of the major peak with -1 and +1 indicating flanking peaks having one less or one more G residue. Horizontal line, average for relative area under the peak; n, number of colonies.

In order to further investigate how repeat number influences slippage during PCR, we determined the areas for the main and flanking peaks in analyses of single colonies from a single 96-well plate (see S2 File). Single colonies are expected to have low numbers of phase variants as the previously measured PV rates indicate that phase variants would, on average, be generated at a frequency of <0.05 per colony for tracts of G8 to G12 [8]. The area under the flanking peaks was found to increase from 14% with G8 tracts to 39% with G11 and to increase as a function of repeat number (Fig 3A). This correlation was consistent both between different PV loci (Fig 3B) and between different tract lengths (S2 Table) of the same gene (Two-way ANOVA, p<0.001). This indicates that repeat number but not flanking sequence is the main determinant of switching rate. As the switching frequencies were determined to be 0.004 for a G8 tract in *cj1139* and 0.02 for a G11 tract in *capA* [8], these results indicate that analysis of a mixed population will tend to overestimate the frequencies of phase variants by 20–35 fold for all repeat numbers.

### General applicability of the PV analysis assay

In order to assess whether these primer sets would have utility for analysis of SSR in other *C*. *jejuni* strains, we performed a BLAST analysis of primer conservation in six additional genome sequences (S3 Table). The current primer sets would work or need minor adjustments on 4–12 phase-variable loci per genome whereas other genes would require new primer sets. Primer design is however relatively straight-forward and could be rapidly extended to other strains. The PSAnalyse program would work on any other similar data set and would not require any adaptation. The only requirement would be determination of repeat number for each locus (from individual sequences of each locus for a control DNA sample of the specific strain) and expression states (obtained by analysis of either a whole genome sequence or of individual gene sequences). This data set would enable derivation of a calibration file. The Peakset data could then be generated by running fragment analysis assays with the control DNA sample to determine the association between fragment size and repeat number.

The PV analysis assay is also applicable to a range of SSR unit sizes. The only limiting factor is whether or not PCR products overlap in size and fluorescence dye type. Larger unit sizes such as penta- and tetranucleotide repeats will exhibit variation over a larger range of sequence space limiting the number of loci that can be incorporated into an assay. There is, however, the possibility of modifying this program to analyse data outputs for microsatellite STR patterns.

In summary, the PV analysis assay and the computer program, PSAnalyse, described here have general applicability for determining repeat number and associated expression state in any organism wherein this phenomenon is observed.

## Supporting Information

S1 Fig Comparison of single colony and total population analyses of the % ON state for individual genes of a *Campylobacter jejuni* strain NCTC11168 population. Bacterial populations were obtained by plating serial dilutions of caecal samples from chickens infected for 52 days with *C*. *jejuni* strain NCTC11168H (Lango-Scholey *et al*., unpublished data). The total population is a sweep obtained from a low dilution plate while single colonies were obtained from high dilution plates. The displayed values represent the difference between the % ON values for the total population (as determined from the fragment analysis by dividing the area of the peaks for an ON number of repeats by the total area under all peaks) and multiple colony analysis (as determined from an analysis of between 11 and 30 colonies with the number of colonies with an ON repeat number being divided by the total number of colonies analysed) as obtained for each gene from 16 individual birds. The top panel shows the genes in which the majority of the population was in the ON state while the bottom panel shows the majority OFF state genes. Average, mean %ON state for a gene. Tracts, repeat number of major peak observed in total population analysis (note that not all birds had the same tract length for each gene hence multiple values are obtained). Positive and negative values indicate that the % ON is higher or lower, respectively, in the ‘total DNA’ as compared to the single colony analysis. Pink, negative 16–33% difference; Red, >33% negative difference; Green, 16–33% positive difference; Blue, >33% positive difference.
(PPTX)

S1 File Percentage ON state data for total population analyses. (XLSX)

S2 File Slippage data obtained from single colony analyses. (XLSX)

S1 Table Confirmation of predicted repeat numbers from fragment analysis by dideoxy sequencing of PCR products. (DOCX)

S2 Table Slippage in repeat tracts during single colony analyses. (DOCX)

S3 Table Conservation of primer bindings sites in selected *C*. *jejuni* strains. (DOCX)
