# Supplementary figures and images for: High Throughput Method for Analysis of Repeat Number for 28 Phase Variable Loci of Campylobacter jejuni Strain NCTC11168
Source: PLoS One. 2016 Jul 28;11(7):e0159634. doi: 10.1371/journal.pone.0159634 (PMC4965091; doi:10.1371/journal.pone.0159634)

## Slide 1
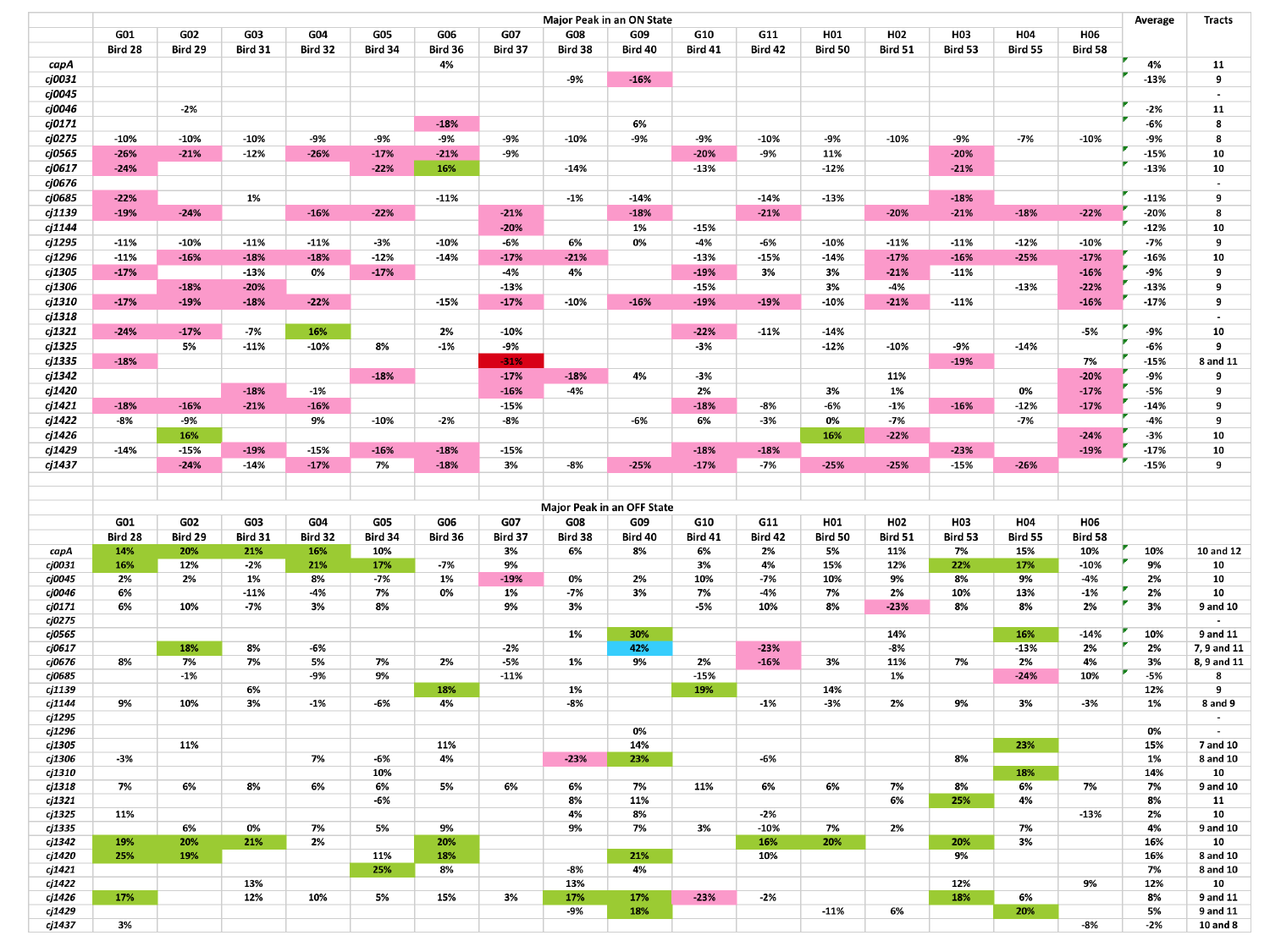

Supplement: S1 Fig — Bacterial populations were obtained by plating serial dilutions of caecal samples from chickens infected for 52 days with C. jejuni strain NCTC11168H (Lango-Scholey et al., unpublished data). The total population is a sweep obtained from a low dilution plate while single colonies were obtained from high dilution plates. The displayed values represent the difference between the % ON values for the total population (as determined from the fragment analysis by dividing the area of the peaks for an ON number of repeats by the total area under all peaks) and multiple colony analysis (as determined from an analysis of between 11 and 30 colonies with the number of colonies with an ON repeat number being divided by the total number of colonies analysed) as obtained for each gene from 16 individual birds. The top panel shows the genes in which the majority of the population was in the ON state while the bottom panel shows the majority OFF state genes. Average, mean %ON state for a gene. Tracts, repeat number of major peak observed in total population analysis (note that not all birds had the same tract length for each gene hence multiple values are obtained). Positive and negative values indicate that the % ON is higher or lower, respectively, in the ‘total DNA’ as compared to the single colony analysis. Pink, negative 16–33% difference; Red, >33% negative difference; Green, 16–33% positive difference; Blue, >33% positive difference. (PPTX) [file pone.0159634.s001.pptx]
