# Supplementary material for: High Throughput Method for Analysis of Repeat Number for 28 Phase Variable Loci of Campylobacter jejuni Strain NCTC11168
Source: PLoS One. 2016 Jul 28;11(7):e0159634. doi: 10.1371/journal.pone.0159634 (PMC4965091; doi:10.1371/journal.pone.0159634)
Supplement: S1 Table — (DOCX) [file pone.0159634.s004.docx]

S1 Table. Confirmation of predicted repeat numbers from fragment analysis by dideoxy sequencing of PCR products

| **Gene/locus** | **Colony name or source plate** | **Sample location in 96-well plate** | **Expected tract from fragment analysis** | **Tract size from sequencing** |
| --- | --- | --- | --- | --- |
| *cj0031* | conA | multiple^1^ | G10 | 10 |
|  | H2 | multiple | G9 | 9 |
| *cj0045* | conA | multiple | G9 | 9 |
|  | H2 | multiple | G10 | 10 |
| *cj0046* | III | A07 | G14 | 14 |
|  | III | A08 | G11 | 11 |
|  | III | A09 | G10 | 10 |
|  | III | A10 | G13 | 13 |
|  | XV | D01 | G10 | 10 |
|  | XV | D02 | G11 | 11 |
|  | XV | E11 | G9 | 9 |
|  | XV | H05 | G10 | 10 |
|  | XV | H06 | G10 | 10 |
|  | conA | multiple | G10 | 10 |
| *cj0171* | conA | multiple | G9 | 9 |
|  | H2 | multiple | G9 | 9 |
| *cj0275* | conA | multiple | G8 | 8 |
|  | H2 | multiple | G8 | 8 |
| *cj0565* | conA | multiple | G10 | 10 |
|  | H2 | multiple | G10 | 10 |
| *cj0617* | conA | multiple | G10 | 10 |
|  | H2 | multiple | G9 | 9 |
| *capA (cj0628)* | VIII | A10 | G12 | 12 |
|  | VIII | C10 | G11 | 11 |
|  | VIII | F10 | G10 | 10 |
|  | XII | A07 | G10 | 10 |
|  | XII | D07 | G11 | 11 |
|  | XII | G07 | G12 | 12 |
|  | conA | multiple | G10 | 10 |
|  | H2 | multiple | G10 | 10 |
| *cj0676* | conA | multiple | G11 | 11 |
|  | H2 | multiple | G9 | 9 |
| *cj0685* | XII | A01 | G9 | 9 |
|  | XII | B01 | G8 | 8 |
|  | XX | B10 | G9 | 9 |
|  | XX | C10 | G10 | 10 |
|  | XX | F10 | G8 | 8 |
|  | conA | multiple | G8 | 8 |
|  | H2 | multiple | G8 | 8 |
| *cj1139* | conA | multiple | G8 | 8 |
|  | H2 | multiple | G8 | 8 |
| *cj1144* | conA | multiple | G8 | 8 |
|  | H2 | multiple | G9 | 9 |
| *cj1295* | XII | A01 | G10 | 10 |
|  | XII | B01 | G9 | 9 |
|  | XII | A10 | G10 | 10 |
|  | XII | A11 | G9 | 9 |
|  | XX | D05 | G8 | 8 |
|  | XX | A10 | G10 | 10 |
|  | conA | multiple | G9/G10 | 9&10 |
|  | H2 | multiple | G9 | 9 |
| *cj1296* | conA | multiple | G9 | 9 |
|  | H2 | multiple | G9 | 9 |
| *cj1305* | conA | multiple | G9 | 9 |
|  | H2 | multiple | G9 | 9 |
| *cj1306* | conA | multiple | G8 | 8 |
|  | H2 | multiple | G8 | 8 |
| *cj1310* | VIII | A10 | G10 | 10 |
|  | VIII | B10 | G9 | 9 |
|  | VIII | F03 | G11 | 11 |
|  | XII | D02 | G10 | 10 |
|  | XII | E02 | G9 | 9 |
|  | XV | F07 | G11 | 11 |
|  | XV | F08 | G9 | 9 |
|  | XV | F09 | G10 | 10 |
|  | XV | H05 | G10 | 10 |
|  | XV | H06 | G10 | 10 |
|  | conA | multiple | G10 | 10 |
|  | H2 | multiple | G10 | 10 |
| *cj1318* | conA | multiple | G9 | 9 |
|  | H2 | multiple | G10 | 10 |
| *cj1321* | conA | multiple | G10 | 10 |
|  | H2 | multiple | G11 | 11 |
| *cj1326* | conA | multiple | G10 | 10 |
|  | H2 | multiple | G10 | 10 |
| *cj1335* | conA | multiple | G10 | 10 |
|  | H2 | multiple | G9 | 9 |
| *cj1342* | conA | multiple | G9 | 9 |
|  | H2 | multiple | G10 | 10 |
| *cj1420* | conA | multiple | G9 | 9 |
|  | H2 | multiple | G9 | 9 |
| *cj1421* | XV | H05 | G9 | 9 |
|  | XV | H06 | G9 | 9 |
|  | conA | multiple | G9 | 9 |
|  | H2 | multiple | G9 | 9 |
| *cj1422* | conA | multiple | G9 | 9 |
|  | H2 | multiple | G9 | 9 |
| *cj1426* | conA | multiple | G10 | 10 |
|  | H2 | multiple | G10 | 10 |
| *cj1429* | conA | multiple | G10 | 10 |
|  | H2 | multiple | G10 | 10 |
| *cj1437* | XV | F07 | G9 | 9 |
|  | XV | G07 | G10 | 10 |
|  | XV | B01 | G9 | 9 |
|  | XV | A01 | No peak | 9 |
|  | XV | F02 | G10 | 10 |
|  | XV | F03 | G9 | 9 |
|  | XV | F04 | G9 | 9 |
|  | XV | G06 | G9 | 9 |
|  | XV | G08 | G9 | 9 |
|  | XX | C11 | G10 | 10 |
|  | XX | C12 | G9 | 9 |
|  | conA | multiple | G11 | 11 |
|  | H2 | multiple | G9 | 9 |

^1^Multiple, indicates that a number of samples from different wells were analysed
