# Supplementary material for: High Throughput Method for Analysis of Repeat Number for 28 Phase Variable Loci of Campylobacter jejuni Strain NCTC11168
Source: PLoS One. 2016 Jul 28;11(7):e0159634. doi: 10.1371/journal.pone.0159634 (PMC4965091; doi:10.1371/journal.pone.0159634)
Supplement: S2 Table — (DOCX) [file pone.0159634.s005.docx]

S2 Table. Slippage in repeat tracts during single colony analyses

| **Gene** | **Area of the Major Peak Relative to Flanking Peaks^1^** | | | | |
| --- | --- | --- | --- | --- | --- |
|  | **G8** | **G9** | **G10** | **G11** | **G12** |
| *cj0275* | **90%**  n=90, s.d.=0.8 |  |  |  |  |
| *cj0685* | **84%**  n=79, s.d.=4.5 | **82%**  n=13, s.d.=0.8 |  |  |  |
| *cj1139* | **83%**  n=77, s.d.=2.8 | **80%**  n=13, s.d.=7.1 |  |  |  |
| *cj1144* |  | **77%**  n=87, s.d.=2.8 |  |  |  |
| *cj1295* |  | **82%**  n=36, s.d.=5.8 | **68%**  n=52, s.d.=2.1 |  |  |
| *cj1310* |  | **80%**  n=35, s.d.=6.0 | **68%**  n=55, s.d.=1.1 |  |  |
| *cj1422* |  | **80%**  n=30, s.d.=1.1 | **67%**  n=51, s.d.=3.6 | **54%**  n=4, s.d.=0.5 |  |
| *cj1321* |  |  | **75%**  n=79, s.d.=2.6 | **64%**  n=10, s.d.=1.0 |  |
| *cj1426* |  |  | **72%**  n=84, s.d.=1.0 | **63%**  n=6, s.d.=2.0 |  |
| *cj1429* |  |  | **77%**  n=86, s.d.=1.2 |  |  |
| *cj0628* (*capA*) |  |  | **74%**  n=24, s.d.=11.2 | **62%**  n=57, s.d.=4.8 | **63%**  n=9, s.d.=0.7 |
| **Average** | **86%**  n=246, s.d.=4.3 | **79%**  n=214, s.d.=4.7 | **72%**  n=431, s.d.=4.9 | **62%**  n=77, s.d.=4.6 | **63%**  n=9, s.d.= 0.7 |

^1^The percentage area under the major peak is determined by dividing the area under the major peak by the combined areas under all the peaks and multiplying by 100; n, number of colonies; sd, standard deviation of all measurements.
