# Supplementary material for: High Throughput Method for Analysis of Repeat Number for 28 Phase Variable Loci of Campylobacter jejuni Strain NCTC11168
Source: PLoS One. 2016 Jul 28;11(7):e0159634. doi: 10.1371/journal.pone.0159634 (PMC4965091; doi:10.1371/journal.pone.0159634)
Supplement: S3 Table — (DOCX) [file pone.0159634.s006.docx]

S3 Table. Conservation of primer bindings sites in selected *C. jejuni* strains

| Gene | Primer  /Length | Strain (ST/BIGSdb id) | | | | | | |
| --- | --- | --- | --- | --- | --- | --- | --- | --- |
|  |  | NCTC11168  (ST-21, 48) | 81116  (ST-283, 1220) | 81-176  (ST-42, 1481) | IA3902  (ST-21, 5293) | RM1221  (ST-354, 18182) | S3  (ST-354, 18183) | M1  (ST-45, 18184) |
|  |  | Number of Identical Nucleotides and PV Status for Each Locus^1^ | | | | | | |
| *cj0031* | Fwd/20 | 20/14* | 20/14* | 20/14* | 20/14* | 20/14* | 20/14* | 20/14* |
|  | Rev/20 | 20 | - | 20 | 16 | 20 | 20 | - |
|  |  | PV |  | not PV | not PV | not PV | PV |  |
| *cj0045* | Fwd/21 | 21 | 21/17 | 21 | 21 | 21 | 21/17 | 21 |
|  | Rev/21 | 21 | 21/16 | 16 | 21 | 21 | 21 | 21/16 |
|  |  | PV | PV | PV | PV | PV | PV | PV |
| *cj0046* | Fwd/20 | 20 | 20 | 20/17* | 20 | 20 | 20/17* | 20 |
|  | Rev/18 | 18 | 18 | 18 | 18 | 18 | 18 | 18 |
|  |  | PV | not PV | PV | G5 | not PV | not PV | not PV |
| *cj0171* | Fwd/20 | 20 | 17 | 20 | 20 | - | - | - |
|  | Rev/20 | 20/14 | - | 20 | 20/14 | 14 | 14 | - |
|  |  | PV |  | PV | PV |  |  |  |
| *cj0275* | Fwd/20 | 20 | 20 | 20 | 20 | 20 | 20 | 20 |
|  | Rev/20 | 20/14 | 17 | 17 | 20/14 | 20/14 | 20/14 | 17 |
|  |  | PV | G5 | G5 | PV | G7 | G7 | G5 |
| *cj0565* | Fwd/21 | 21 | 21 | 20 | 21 | 21 | 21 | 21 |
|  | Rev/20 | 20 | - | - | 20 | - | - | - |
|  |  | PV |  |  | PV |  |  |  |
| *cj0617* | Fwd/20 | 20/14 | 14 | 20/14 | 14 | 20/14 | 20/14 | 14 |
|  | Rev/20 | 20 | 17 | 20/17 | - | 20 | 20 | 17 |
|  |  | PV |  | PV |  | PV | PV |  |
| *capA* | Fwd/20 | 20 | - | - | - | - | - | - |
|  | Rev/20 | 20 | - | - | - | - | - | 14 |
|  |  | PV |  |  |  |  |  |  |
| *cj0676* | Fwd/20 | 20 | 19 | 20 | 20 | 20 | 20 | 20 |
|  | Rev/22 | 22 | 22 | 22 | 22 | 22 | 22 | 22 |
|  |  | G5-PV(G9) | G5-G5 | G5-G5 | PV(G10)-G5 | PV(G10)-G5 | PV(G9)-G5 | G5-G5 |
| *cj0685* | Fwd/25 | 25/15* | 25/15* | 25/15* | 25/15* | 25/15* | 25/15* | 25/15* |
|  | Rev/21 | 21/14* | 21/14* | 21/14* | 21/14* | 21/18*/14* | 21/18*/14* | 21/14* |
|  |  |  | PV | PV | PV | PV | PV | PV |
| *cj1139* | Fwd/18 | 18/15* | 15* | 15* | 18/15* | 15* | 15* | 15* |
|  | Rev/25 | 25 | - | - | 25 | - | - | - |
|  |  | PV |  |  | PV |  |  |  |
| *cj1144* | Fwd/16 | 16 | - | - | 16 | 14 | 14 | - |
|  | Rev/16 | 16 | - | - | 16 | - | - | - |
|  |  | PV |  |  | PV |  |  |  |
| *cj1295* | Fwd/20 | 20 | 18 | 20 | - | 19 | 19 | 18 |
|  | Rev/20 | 20 | 17 | 18 | 20 | 17 | 17 | 17 |
|  |  | PV | PV | PV |  | PV | PV | PV |
| *cj1296* | Fwd/20 | 20 | - | - | 19 | 14 | - | - |
|  | Rev/20 | 20 | 19 | 19 | 20 | 19 | 19 | 19 |
|  |  | PV |  |  | PV |  |  |  |
| *cj1305* | Fwd/24 | 24/19/  18* | 21/18* | 24/24/  18* | 19/19* | 19/19* | 19/19* | 21/18* |
|  | Rev/20 | 20/18 | - | 18 | 19/19 | 18 | 18 | - |
|  |  | PV | PV | PV | not PV | not PV | not PV | PV |
| *cj1306* | Fwd/20 | 20 | - | - | - | 20 | 20 | - |
|  | Rev/19 | 19 | - | - | - | 19 | 19 | - |
|  |  | PV |  |  |  | PV | PV |  |
| *cj1310* | Fwd/22 | 22 | - | - | 22 | 22 | 22 | - |
|  | Rev/21 | 21/16 | - | - | 21/16 | 16 | 16 | 16 |
|  |  | PV |  |  | G6 | PV | PV | not PV |
| *cj1318* | Fwd/22 | 22 | 22 | 19 | 19 | 22 | 22 | 22 |
|  | Rev/20 | 20/20 | - | - | 20/20 | - | - | - |
|  |  | PV |  |  | PV |  |  |  |
| *cj1321* | Fwd/20 | 20 | - | 14* | - | 15* | 15* | - |
|  | Rev/20 | 20 | - | - | - | - | - | - |
|  |  | PV |  |  |  |  |  |  |
| *cj1326* | Fwd/22 | 22/19 | - | 19 | 19/19 | - | - | - |
|  | Rev/21 | 21 | - | - | - | 14 | - | - |
|  |  | PV |  |  |  |  |  |  |
| *cj1335* | Fwd/20 | 20 | - | - | 20 | 20 | 20 | - |
|  | Rev/20 | 20/20 | - | - | 20/20 | - | - | - |
|  |  | PV |  |  | PV |  |  |  |
| *cj1342* | Fwd/20 | 20 | 19* | 16* | 19* | 19* | 19* | 19* |
|  | Rev/20 | 20/15 | - | 20 | 19/15 | 19/15 | 19/15 | - |
|  |  | PV | PV | PV | PV | PV | PV | PV |
| *cj1420* | Fwd/20 | 20 | 19 | 20 | 19 | - | - | 19 |
|  | Rev/20 | 20 | 20 | 20 | 20 | - | - | 20 |
|  |  | PV | PV | PV | PV |  |  | PV |
| *cj1421* | Fwd/22 | 22/22 | - | - | - | - | - | - |
|  | Rev/25 | 25/15* | 15/15* | 15/15* | 15* | 15 | 15* | 15/15* |
|  |  | PV |  |  |  |  |  |  |
| *cj1422* | Fwd/22 | 22/22 | - | - | - | - | - | - |
|  | Rev/21 | 21 | - | - | - | - | - | - |
|  |  | PV |  |  |  |  |  |  |
| *cj1426* | Fwd/18 | 18 | - | - | - | - | - | - |
|  | Rev/20 | 20 | - | - | - | - | - | - |
|  |  | PV |  |  |  |  |  |  |
| *cj1429* | Fwd/20 | 20 | 14* | 19* | 15 | 15 | 15 | - |
|  | Rev/20 | 20 | - | - | - | - | - | - |
|  |  | PV |  |  |  |  |  |  |
| *cj1437* | Fwd/20 | 20 | - | - | - | - | - | - |
|  | Rev/20 | 20 | - | - | - | - | - | - |
|  |  | PV |  |  |  |  |  |  |

^1^For each oligonucleotide the number of identical nucleotides is indicated as obtained in a BlastN search performed in CampyMLST BIGSdb (note short matches may not be in the indicated locus). Additional matching sequences are indicated after the back slash. An asterisk (*) indicates that the 3’ nucleotide does not match. A dash (-) indicates that only matches of <14 nucleotides were obtained or that two nucleotides at the 3’ end of the oligonucleotide were not matching. PV, phase variation possible as a tract of G8 or longer is present in this locus; not PV, no G-tract present in the locus. Cyan, primer binding sites completely conserved and locus is subject to PV; yellow, primer binding site is partially conserved or there is a potential for non-specific binding of primers but locus is subject to PV; grey, primer binding sites are completely or partially preserved but locus is not subject to PV.
